# Supplementary figures and images for: Community Antibiotic Consumption in Cyprus for the Period 2015 to 2022
Source: Antibiotics (Basel). 2024 Jan 4;13(1):52. doi: 10.3390/antibiotics13010052 (PMC10812799; doi:10.3390/antibiotics13010052)

Antibiotic consumption (packages per inhabitant) by year in Cyprus

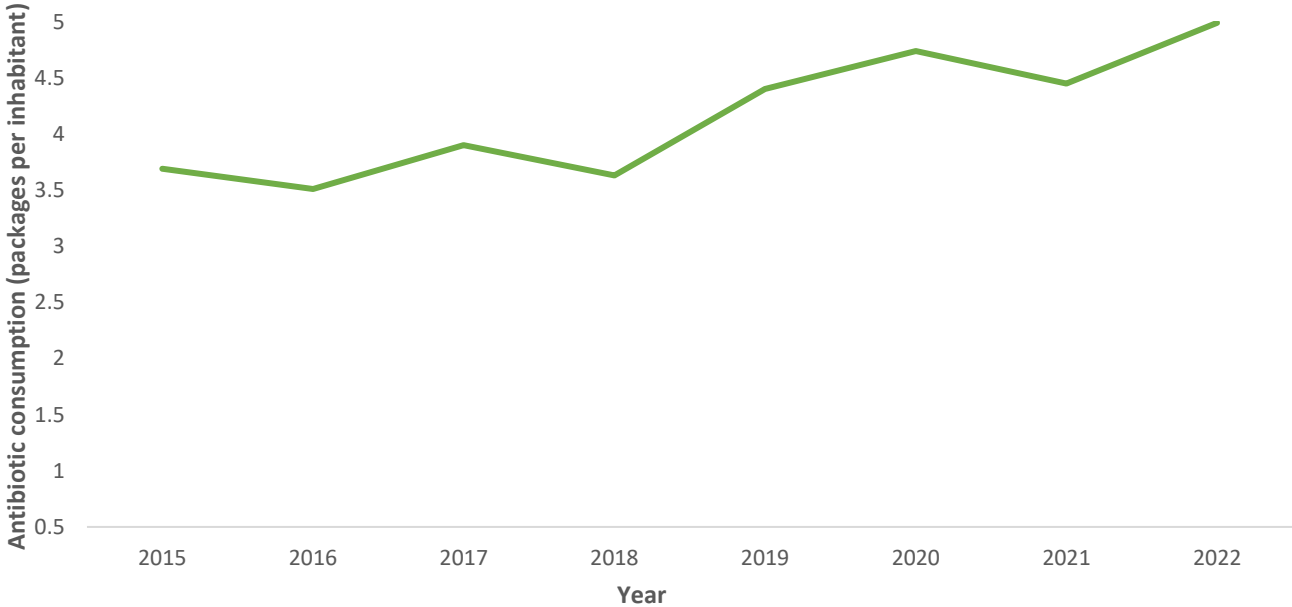

Supplement: Supplementary file 1 [file antibiotics-13-00052-s001.zip › antibiotics-2768232-supplementary.pdf]
